# Parent-implemented early intervention design for improving speech and language skills among Mandarin-speaking infants and toddlers with cleft lip and/or palate

**DOI:** 10.3389/fpubh.2025.1458452

**Published:** 2025-01-20

**Authors:** Sha Lai, Li Lu, Zhongliang Zhou, Chi Shen, Binting Yang, Zhanping Ren, Siwei Ma

**Affiliations:** ^1^School of Public Policy and Administration, Xi’an Jiaotong University, Xi’an, Shaanxi, China; ^2^Key Laboratory of Shaanxi Province for Craniofacial Precision Medicine Research, College of Stomatology, Xi’an Jiaotong University, Xi’an, Shaanxi, China; ^3^Department of Speech and Language Disorders, College of Stomatology, Xi’an Jiaotong University, Xi’an, Shaanxi, China; ^4^Department of Cleft Palate-Craniofacial Surgery, College of Stomatology, Xi’an Jiaotong University, Xi’an, Shaanxi, China

**Keywords:** language skills, cleft lip and/or palate, infants and toddlers, intervention, intervention mapping

## Abstract

**Background:**

Cleft lip and/or palate (CL/P) speech is a significant communication disorder with notable and enduring consequences. This study focused on developing an intervention mapping (IM)-based and parent-implemented early intervention to improve speech and language skills among infants and toddlers with CL/P in China.

**Methods:**

An IM procedure was used to develop a parent-implemented early intervention. First, parent–child interaction problems affecting language development in infants and toddlers were identified through 132 questionnaires completed by parents of children with CL/P and 30 family videos of parent–child interactions. Second, according to the problem diagnosis, the logical model of parent–child interaction behavior change was constructed. Finally, the health outcomes were transformed into intervention goals at three levels (i.e., infants and toddlers with CL/P, parents and environment), and a set of early intervention programs were designed by integrating various intervention techniques.

**Results:**

The diagnosis of parent–child interaction problems showed that 40.91% of parents and children often use electronic media together; in parent–child interaction, 41.67% of parents presented ‘lack of waiting’, 29.55% overcorrected pronunciation errors. Video analysis revealed issues in parent–child interaction, such as inadequate parental skills, ineffective interactions, and an unsuitable environment. A parent-implemented early intervention was developed, including the following specific steps: health education within the hospital, 9 days of an online reading program in WeChat groups, face-to-face standardized training workshops, and individualized video feedback therapy.

**Conclusion:**

We have devised a parent-implemented early intervention to enhance speech and language abilities in Mandarin-speaking infants and toddlers with CL/P, overcoming the shortage of speech therapy services in China.

## 1 Introduction

Cleft lip and/or palate (CL/P) is a prevalent congenital anomaly with a global live birth incidence rate of 1.08/1,000 (1). In China, the incidence is reported to be 1.48–3.27/1,000 live births (2, 3), which is notably higher than in other regions, such as North America (1.56/1,000), Europe (1.55/1,000), Oceania (1.33/1,000), South America (0.99/1,000), and Africa (0.57/1000) (4, 5).

Research has revealed that cleft palate speech is a significant communication disorder with notable and enduring consequences. Studies indicate that 36–47 month-old toddlers with cleft lip and palate experience a staggering 69.5% delay in vocabulary skills (6). Additionally, 8–16 month-old Mandarin-speaking infants with cleft lip and palate experience an 85% delay in expressive vocabulary skills (7). By age 3, approximately 50% of children with CL/P exhibit noticeable phonological and articulation disorders, which might persist throughout their preschool years (8, 9). Unfortunately, early language delays often persist into the teenage and adult years, resulting in social, emotional, and learning challenges that contribute to higher rates of poor mental health and unemployment (10). Since individual responses to treatment can vary significantly based on craniofacial morphology, these difficulties persist over time and can seriously impact the well-being of CL/P children and their families (11).

It is widely and internationally acknowledged that early intervention to promote speech and language development is crucial. Some of the most influential intervention techniques include the “It Takes Two to Talk” program in Canada, the “Language is Key” program in the United States, the Focused Stimulation Approach program, and the Enhanced Milieu Teaching program. However, there is a notable lack of interventional studies on delayed speech and language development in infants with CL/P, such as the Milieu Teaching program (12) and the Enhanced Milieu Teaching with Phonological Emphasis program (13, 14). Furthermore, the American Speech-Language-Hearing Association’s early intervention guidelines stress the importance of parent-implemented interventions (15).

Language and communication skills in children are primarily cultivated through interactive engagement with their primary caregivers. Active parent–child interaction constitutes a highly effective framework for facilitating language acquisition (16–18). These interactions are inherently reciprocal, characterized by mutual responsiveness and dynamic feedback (19, 20). However, the trajectory of language development is contingent upon various factors, including the child’s individual growth patterns, the caregiver’s competencies, the quality of interactive exchanges, and the surrounding environmental influences, which can either enhance or hinder this process (20). From a mechanistic standpoint, enriched parent–child interactions play a pivotal role in supporting neural plasticity, a fundamental neurobiological substrate for early learning. This plasticity underpins the acquisition of a range of developmental competencies, with language skills being a critical component (21). Research underscores a robust association between positive parenting practices, high-quality parent–child interaction, and the progressive development of linguistic capabilities in children (16, 18, 22). In particular, for infants and young children experiencing language delays, naturalistic interactions alone often fall short in providing the necessary support for effective language acquisition (23). Meta-analytical findings suggest that the linguistic environment constructed by caregivers of children with language delays is frequently less supportive than that observed in typically developing peers. This disparity manifests in reduced interaction frequency, diminished quality of feedback, and lower richness and quantity of language input (24).

Parent-implemented interventions (also commonly known as home programs) are vital intervention methods for children with delayed language development, and they stand out for their emphasis on higher parent involvement than traditional therapy across various countries and service backgrounds (10). Home programs are available in diverse forms, including parent training, remote consultation, use of commercial products, direct intervention with supervision, and real-time communication with a speech-language pathologist. However, despite their popularity, research on parent involvement is limited (25–27), with inadequate specific detail on how parents can implement these interventions (24, 28), with an overemphasis on the techniques that promote language development. Currently, there is no definitive recommendation regarding the optimal intervention time, duration, mode, or frequency of these programs (29, 30). Therefore, more robust studies that thoroughly evaluate home programs and better clarify how parents can effectively implement interventions are necessary to enhance their efficacy in assisting children with delayed language development (31).

Phonetic differences exist between Mandarin and English, specifically in consonants, vowels, and syllable structures. Furthermore, social and cultural factors might influence infants’ speech and language development. As a result, caution should be exercised when considering intervention programs in a Mandarin context. With 848 million Mandarin speakers worldwide and the largest proportion of CL/P patients, it is vital to prioritize early intervention. A “parental involvement” approach has emerged as an effective solution due to the limited availability of speech/language pathologists in China. Therefore, it is urgent to develop culturally appropriate early intervention programs for Mandarin-speaking CL/P patients.

Intervention mapping (IM) is a widely used planning framework for health promotion programs that offers a systematic and evidence-based approach to identifying problems and developing effective solutions (32). It has been applied successfully in diverse healthcare settings, such as community and clinical environments, to enhance health education and promote interventions (33–36). However, its use in designing interventional programs in speech–language pathology remains limited.

This study focuses on the IM approach to developing speech and language skills among Mandarin-speaking infants and toddlers with CL/P via parent-implemented early intervention. The implementation of parent-implemented early intervention can overcome barriers preventing access to professional services provided by speech–language therapists/pathologists. This article mainly analyzed the problem diagnosis of early intervention by parents, determination of outcomes and change objectives, selection of methods and practical strategies and formulation of the implementation plan based on IM approach.

## 2 Methods

### 2.1 Study setting and populations

This study was conducted at the CL/P Center of the Stomatological Hospital of Xi’an Jiaotong University, the largest treatment center in northwestern China, which provides sequential treatment for patients with CL/P from across the country, particularly in the northwestern regions. The hospital has an annual outpatient volume of nearly 700,000 visits, performing approximately 500 surgeries and managing 1,000 CL/P-related speech therapy cases annually. The study population consisted of infants and toddlers with CL/P and their parents who met the inclusion and exclusion criteria. Inclusion criteria for infants and toddlers in both groups included: (1) no prematurity (premature delivery was defined at ≤37 w), (2) no low body weight (low body weight was defined at ≤5,000 g), (3) no congenital systemic diseases, (4) no congenital hypoxia, (5) no syndromic conditions, (6) passing growth and development screening, and (7) receiving CL/P surgery at 8–16 months. Exclusion criteria included: (1) complications after CL/P surgery accompanied by visible palatal fistula, and (2) systemic diseases unrelated to CL/P with growth and development.

### 2.2 Study design and procedures

**Figure 1:**
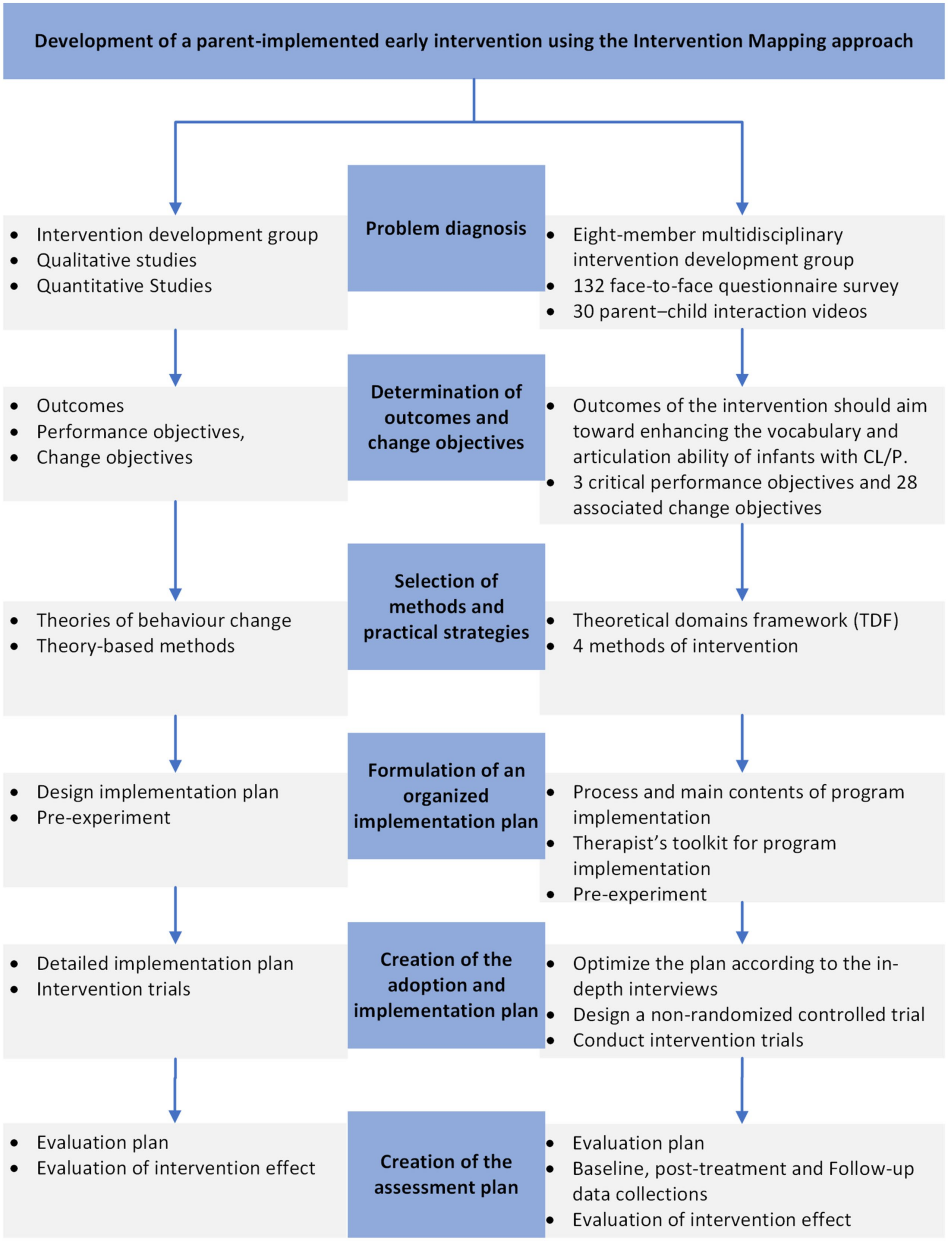
Flow chart of development a parent-implemented early intervention using the intervention mapping approach.

IM was implemented through six steps: (1) problem diagnosis; (2) determination of outcomes and change objectives; (3) selection of methods and practical strategies; (4) formulation of the implementation plan; (5) adoption and implementation; (6) creation of the assessment plan (Figure 1).

### 2.3 Step 1: Problem diagnosis

#### 2.3.1 Intervention development group

An eight-member multidisciplinary intervention development group was formed, including two parents of infants with CL/P, one speech–language pathologist, two speech–language therapists, one health intervention expert, one psychological and behavioral expert, and one parent coordinator. The group, led by a speech–language pathologist with a senior title, met monthly for 3 months to have discussions focusing on speech and language development in infants with CL/P, family intervention, and any obstacles and enablers for intervention development. Additionally, the group addressed the intervention program’s outcomes, delivery, practical strategies, and feasibility.

#### 2.3.2 Problem analysis

In our earlier study, we have studied the status and associated factors of delayed speech and language development in infants and toddlers with CL/P (7, 37, 38), and found that the delays in receptive and expressive vocabulary skills in Mandarin-speaking infants and toddlers with CL/P was common, and age was positively correlated with impaired vocabulary ability and hysteresis, parent–child interaction hours (>3 h/day) was negative correlation with delays in receptive vocabulary skills, suggesting the necessity and importance of early and effective intervention. Therefore, the problem diagnosis focused on parent–child interactions in the family and identified them based on the theoretical domains framework (TDF).

We randomly selected infants and toddlers with CL/P aged 8 to 16 months who met both inclusion and exclusion criteria from their surgical medical records within 18 months and conducted a face-to-face questionnaire survey to their primary caregivers (i.e., primary caregivers can be either mother or father). The questionnaire was designed based on TDF and consisted of knowledge, skills, social responsibility, beliefs in their ability and outcomes, objectives, motivations, emotions, and environmental background and resources related to parent–child interaction on language development. We distributed 134 questionnaires to primary caregivers of patients and 132 valid were collected. The questionnaire in this study had a Cronbach’s alpha of 0.82.

Video observational analysis was used to evaluate the problems in parent–child interactions in the family setting. 34 primary caregivers of Infants and Toddlers with CL/P, randomly selected from 132 who completed the questionnaires, were asked to provide a 5–6 min parent–child interaction video with the informed consent of their primary caregiver. The video is required to be everyday scenes in the home environment with language interaction between primary caregivers and children. Totally, 30 parent–child interaction videos have been gathered. The primary aim of this observational study was to examine parent–child interactions during communication by scrutinizing parents’ unconscious behaviors toward speech and language input, as well as the infant’s reactions, and then used to formulate the intervention design. The videos underwent category analysis using the problem-focused analysis to assess and identify frequently recurrent interaction behaviors unfavorable for speech and language development. Similar problems were identified, categorized, and named based on certain concepts, such as “lack of waiting” and “speak quickly.” Two therapists with more than 5 years of speech therapy experience recorded the following 3 aspects through video observation independently: interaction skills, interaction effect, and interaction environment.

### 2.4 Step 2: Determination of intervention outcomes, performance objectives, and change objectives

The intervention development group concurred that the outcomes of the intervention should aim toward enhancing the vocabulary and articulation ability of infants with CL/P. Moreover, performance objectives and their corresponding change objectives were specified at the individual, parent/caregiver, and environmental levels using the socio-ecological model. A matrix of specific intervention objectives was created through a deterministic approach at the intersection of determinants and performance objectives. This method facilitated the creation of a set of change objectives, which served as a cohesive action statement to describe the intervention’s parameters.

### 2.5 Step 3: Selection of methods and practical strategies

The third step in the IM process entailed identifying theory-based intervention methods, including intervention programs and strategies known to influence variable factors and circumstances conducive to change. These theoretical methods were then adapted into practical strategies that aligned with the change objectives outlined in the matrix.

### 2.6 Step 4: Development of the program

All information from the previous steps was combined in the fourth step. The behavioral change theory was linked with intervention measures to design program implementation strategies and prepare materials needed to implement the intervention. Considering demographic and cultural differences, the intervention program was pre-tested in 30 infants’ and toddlers’ family that completed the questionnaires and parent–child interaction videos to ensure its feasibility.

### 2.7 Step 5: Adoption and implementation intervention plan

An implementation intervention plan was developed in consultation with the intervention development group. In this step, the intervention plan was modified and improved by collecting feedback from speech therapists and pre-trial participants, and a non-randomized controlled trial was designed given ethical concerns in interventions in infants and toddlers with CL/P.

### 2.8 Step 6: Creation of the assessment plan

After completing the previous steps, the assessment plan was created to evaluate the process and effects of the intervention, ensuring that the objectives defined in Steps 2 were attained.

All procedures performed in studies involving human participants were in accordance with the ethical standards of the Research Ethics Committee of Xi’an Jiaotong University (2021–1150) and with the 1964 Helsinki declaration and its later amendments or comparable ethical standards.

## 3 Results

### 3.1 Step 1: Problem diagnosis

A total of 132 infants and toddlers with CL/P were included in problem diagnosis, and 50.8% of them were girls (Table 1). Table 2 showed the knowledge, skills, roles, beliefs, motivations, emotions and circumstances of parents in improving speech skills in infants and toddlers with CL/P. Our investigation found that there is a lack of knowledge and skills among primary caregivers in promoting children’s language development. Almost 40.91% of parents often used electronic screens with their children, while 37.12% of parents exhibited a deficiency in patience during communication. Furthermore, nearly 29.55% of parents tended to overcorrect (i.e., every time) their children’s errors in pronunciation. Conversely, the vast majority of parents (98.48%) have the willingness to promote children’s language development.

**Table 1:** Sample characteristics of infants and toddlers with CL/P (*n* = 132).

Variables	Categories	N	%
Sex	Male	65	49.2
	Female	67	50.8
Age in months	9	2	1.5
	10	9	6.8
	11	18	13.6
	12	25	18.9
	13	23	17.4
	14	17	12.9
	15	14	10.6
	16	11	8.3
	17	9	6.8
	18	4	3.0
Mather’s education	High school and below	102	77.3
	College and above	30	22.7
Father’s education	High school and below	100	75.8
	College and above	32	24.2
Household monthly income	Less than 3,000 yuan	59	44.7
	3,000–5,000 yuan	27	20.5
	5,000–10,000 yuan	31	23.5
	10,000 yuan and above	15	11.4
Number of family members living together	2	1	0.8
	3	30	22.7
	4–5	57	43.2
	More than 5	44	33.3

**Table 2:** The knowledge, skills, roles, beliefs, motivations, emotions and environments of parents in improving speech and language skills in infants and toddlers with CL/P (*n* = 132).

Items		Options	N	%
Knowledge	Use electronic media together	Often	54	40.91
		Occasionally	68	51.52
		Never	10	7.58
Skills	Interaction state of children	Positive	90	68.18
		General	42	31.82
	Aware of the interaction problem (multiple-choice questions)	Incessant talking	28	21.21
		Offering help in absence of needs	79	59.85
		Interrupting frequently	10	7.58
		Self- righteous understanding of children	55	41.67
	Teach speaking in interaction	Consciously taught	46	34.85
		Unconscious	86	65.14
	Positional relationships in interactions	Same sight, face to face	100	75.76
		Others	32	24.24
	Frequency of error correction	Every time	39	29.55
		Occasionally	33	25.00
		Only in deliberate practice	25	18.94
		Others	35	26.52
	Tone of error correction	Gentle tone	96	72.73
		Stern tone	36	27.27
Social role and identity	Time spent with father or mother	About the whole day	112	84.85
		Half a day or less	20	15.15
	Frequency of conscious interaction	Frequently	49	37.12
		Occasionally	83	62.88
	Length of conscious interaction	≤3 h	73	55.30
		>3 h	59	44.70
Belief in ability	Perceived role in speech teaching	Important role	120	90.91
		Partial role	12	9.09
		No role	0	0.00
Belief in outcomes	Beliefs toward the consequences of speech	No concerns	21	15.91
		Concerns	78	59.09
		Others	33	25.00
Objectives and motivations	Willingness to upgrade skills	Yes	130	98.48
		No	2	1.52
Emotional state	Patient	Yes	83	62.88
		No	49	37.12
	Strategy when not cooperating	Change tactics and try again	68	51.52
		Persuade or explain	52	39.39
		Take no measures	12	9.09
Environmental background	Preference for professional guidance	Necessary	117	88.64
		Not necessary	15	11.36

Behavioral observations were performed by analyzing video recordings. The results revealed deficiencies in interaction skills within parent–child interactions, including lack of waiting, lack of observation of children’s communication intentions, utilization of age-inappropriate forms of language, fast speech speed, and using imperative utterances more than three times. Furthermore, adverse interaction effects between parent–child interaction behaviors were founded, such as poorly designed play, unsustainable interaction and inattentive attention in children as well as in suboptimal interaction environments marked by noise and inappropriate toys (Table 3).

**Table 3:** Results of behavioral observations based on videos of family parent–child interaction in infants and toddlers with CL/P (*n* = 30).

Items	Problems	Number of cases
Parent–child interaction skills	Lack of waiting	30
	Lack of observation of children’s communication intentions	24
	Utilization of age-inappropriate forms of language	24
	Speak quickly	21
	Utilization of imperative statements more than 3 times	18
Interaction effect	Interactive games are poorly designed (on clear themes, no sense of sequence or repetition)	21
	Unsustainable interaction	18
	Inattentive attention in children	15
Interactive environment	Interactive environmental noise	21
	Inappropriate toys	12

Behavioral observations based on 30 videos of family parent–child interaction in infants and toddlers with CL/P. Each video was 5–6 min long and documented everyday scenes in the home environment with language interaction between primary caregivers and their children. Two therapists with more than 5 years of speech therapy experience recorded the problems.

### 3.2 Step 2: Determination of outcomes, performance objectives, and change objectives

Based on discussions in the intervention development group, the ultimate goals and outcomes of the intervention was set to enhance infants’ consonant inventory while reducing the cleft speech characteristics associated with CL/P. Specific outcomes of intervention were decomposed by adhering to the socio-ecological model (39), examining the pertinent aspects of infants, caregivers, and the broader familial and communal environment (Table 4). Performance objectives were then formulated according to the ultimate goals and outcomes of the intervention. Performance objectives and associated change objectives involved problem diagnosis in step 1, all coordinated by TDF.

**Table 4:** The determination of outcomes of the parent-implemented early intervention.

Level	Specific program outcome
Infants and toddlers with CL/P	There were improvements in consonant inventory and a reduction in cleft speech characteristics of infants and toddlers with CL/P.
Parents	Parents could employ interaction techniques to maintain at least 5 min of interaction. They could also use speech and language demonstration techniques for at least 30 min of interaction every day until the age of 2.5.
Family/community environment	Parents created a family nurture environment, including emotions/responses, physical environment organization, toys/games, and diversified information stimuli. There was also a WeChat-based community support atmosphere, which was composed of experience sharing, popular science knowledge transfer, and peer support.

**Figure 2:**
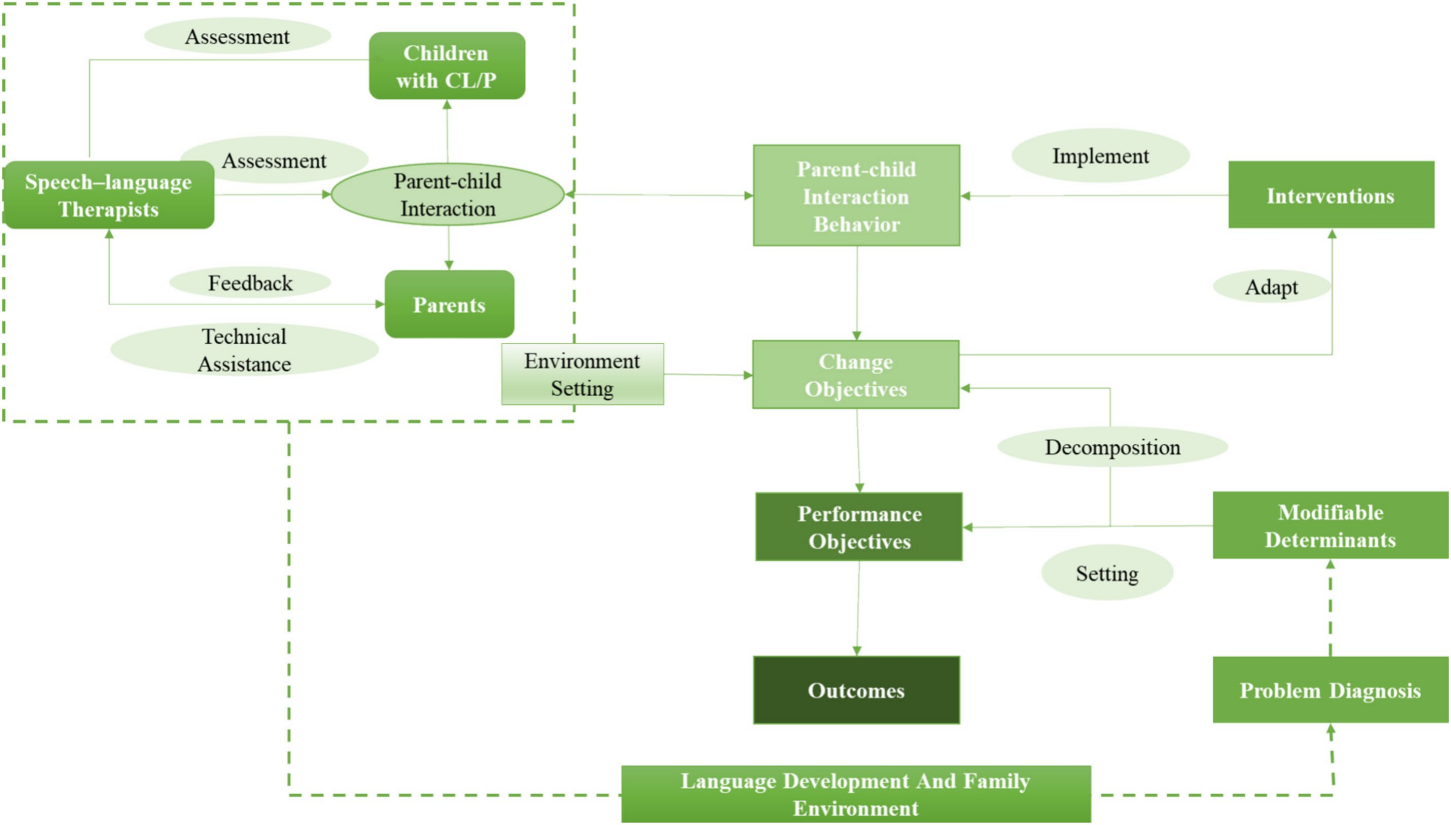
The logic model of the parent-implemented early intervention for developing speech and language among infants and toddlers with CL/P.

As a result, the research group established 3 critical performance objectives and 28 associated change objectives, outlining actionable steps required to achieve the performance objectives (Supplementary Table 1). The results were utilized for development work of the logic model of change and when designing the intervention programs and its contents (Figure 2).

### 3.3 Step 3: Selection of methods and practical strategies

Methods grounded in the theory of behavioral change within the TDF domains were used to facilitate the adoption and maintenance of speech and language development-promoting behaviors. Program implementation strategies and materials were designed by combining this theory with intervention measures. The early intervention program comprised four parts: health education within the hospital, 9 days of an online reading program via groups in WeChat (i.e., the most popular and widely used social media platform in China), face-to-face standardized training workshop, and individualized video feedback therapy. Table 5 shows the intervention strategies. These strategies comprised sharing successful cases in workshops, setting weekly goals in individualized video feedback guidance, and providing detailed explanations and improvement methods for using interactive techniques in individualized video feedback guidance written by the therapist.

**Table 5:** Methods, strategies, implementation materials, and tools used in the early intervention programs.

Determinant	Strategies	Materials/Tools
Individuals		
Knowledge	Provide information	M1. Ask parents to receive health education on CL/P treatment from specialist nurses.
		M2. Distribute leaflets related to early intervention of speech and language rehabilitation in infants with CL/P.
		M3. Follow a WeChat official account spreading popular science knowledge about speech and language rehabilitation in infants with CL/P.
	Assessment & Feedback	M4. Fill in the electronic registration and evaluation form, including Children’s growth and development state, Children’s speech and language development level, Children’s speech and language environment, Parents’ timetable-based awareness.
		M5. Conduct in-hospital group focus interviews.
		M6. The course coordinator would guide everyone to think about problems after learning 3 popular science articles.
Skills	Parents’ training courses	M7. Provide a folder about the organizational process of collective workshop training for parents (for use by the activity leader).
		M8. Organize parents to learn intervention techniques from teaching videos in workshop training courses.
		M9. Provide an electronic folder about early intervention to parents after workshop training courses, including word lists and picture book lists.
	Exercise Feedback & guidance	M10. Provide writing norms on the individualized video feedback guidance scheme based on parent–child interaction (for use by the therapist giving video feedback).
		M11. Provide a checklist of early intervention techniques to be mastered by parents in relation to individualized video feedback guidance based on parent–child interaction (for use by the therapist giving video feedback).
Objective & motivation	Set weekly individualized goals	M12. Write treatment goals in the first row of the feedback record.
	Promote adherence	M13. Share successful cases in workshops.
		M14. Give written praises to parents after they meet the standards on intervention techniques.
	Feedback response	M15. Professional/well-trained therapists give feedback in the hospital focus interview, parents’ collective workshop training course and individualized feedback, and check and confirm the improvement until the stabilization of skills.
Environmental		
Family environment	Training courses	M16. Organize parents to learn the setup of the family environment for parent–child interaction from teaching videos in workshop training courses.
	Exercise Feedback & guidance	M17. Give opinions about the setup of the family environment for parent–child interaction in individualized video feedback guidance according to the realities of various families.
Community environment	Virtual Community	M18. Invite family members to join the WeChat community and participate in collective workshops.
	Community exchanges	M19. Ask a specially-assigned person to take charge of the community reading club, guide positive thinking, and give positive feedback.
	Professional opinions.	M20. Invite surgeons and specialist nurses to speak in collective workshops and provide professional opinions.

M1-M20 stand for intervention materials number.

### 3.4 Step 4: Formulation of an organized implementation plan

First, During the hospitalization of infants with CL/P, their parents would undergo in-hospital focus interviews to educate them on speech and language development and rehabilitate their children. At the end of the interview, they were informed about the purpose and voluntary participation of the intervention program. Voluntary participants would begin early intervention within 3 months after the CL/P surgery. Baseline data and parent–child interaction videos will be collected prior to the intervention.

Second, the early intervention workshop took place monthly, creating a community of participating parents. Face-to-face training sessions were provided at the hospital, with workshop times chosen to accommodate parents’ preferences. Courses “warm-up” involve a 9-day reading program in WeChat groups before a workshop for parents, with the following steps: (1) Notification by phone and creating a WeChat group; (2) organizing community activities within the group, including inviting family members, displaying group rules, attending daily popular science reading activities, raising questions, and reflecting on them; (3) collecting parents’ questions by the course coordinator.

Third, an intervention workshop for parents was designed and delivered, consisting of 3 h of knowledge learning and 6 h of practical workshop training. This included 1 h of group interaction and 5 h of video analysis and feedback. The workshop comprised multiple activities, such as ice-breaking exercises, teaching relevant knowledge, group brainstorming, and skill training. Additionally, there was an opportunity for informal socializing during lunchtime and a “secret” sharing module to provide valuable insight and bonding for participants. At the end of the workshop, learning materials, including “word lists” and “parent–child reading lists,” were distributed, and certificates of completion were issued. By seamlessly linking WeChat groups with the workshop training in this early stage, parents had access to relevant knowledge, skill training programs, and ongoing support.

Fourth, the intervention program centered around individualized video feedback based on parent–child interactions. Families participating in the intervention were asked to provide parent–child interaction videos in five phases to the therapist: at the beginning of the intervention, after receiving workshop training, and then every 2 weeks for a total of three times. The therapist will provide feedback based on intervention techniques and send written guidance via WeChat to the parents.

Fifth, the course administrator sent out relevant popular science knowledge through WeChat every week, and follow up with participating children and their parents every 3 months. Follow-up must be completed when the child is three or three and a half years old.

A comprehensive toolkit was meticulously crafted to facilitate the implementation of a parent-involved early intervention program designed to promote a process-oriented intervention method. This toolkit incorporates a range of plans and materials necessary for program implementation (for a comprehensive list, see Supplementary Table 2), including a series of workshop teaching points, insights on individualized video feedback, and a checklist highlighting sub-objectives for parent behaviors. The intervention program was pre-tested in 30 infants’ and toddlers’ family that completed the questionnaires and parent–child interaction videos to ensure its feasibility.

### 3.5 Step 5: Adoption and implementation plan

In step 5, the goal was to plan for adopting and implementing the intervention study, involving the collaboration of several professionals, including speech–language pathologists and therapists, health experts, and parent representatives. They conducted group focus and in-depth interviews with pre-tested groups, potential implementation organizations and target groups to identify potential barriers and facilitators of program implementation. Feedback from hospital speech–language therapists, parents, and target group representatives were considered, ultimately resulting in modifications and improvements in the intervention plan.

In step 5, a non-randomized controlled trial was designed given ethical concerns in interventions in infants and toddlers with CL/P. Children and parents participated in the intervention voluntarily. The inclusion and exclusion criteria for the intervention and control groups were determined, along with the sample size. Inclusion criteria for infants and toddlers in both groups included: (1) no prematurity (premature delivery was defined at ≤37 w), (2) no low body weight (low body weight was defined at ≤5,000 g), (3) no congenital systemic diseases, (4) no congenital hypoxia, (5) no syndromic conditions, (6) passing growth and development screening, and (7) receiving CL/P surgery at 8–16 months. Exclusion criteria included: (1) complications after CL/P surgery accompanied by visible palatal fistula, and (2) systemic diseases unrelated to CL/P with growth and development.

In accordance with the statistical norms for speech and language development, the intervention will implement at one or one and a half years old. Four major intervention strategies will be completed within 3 months. Four follow-up visits will be conducted every 6 months after the end of the intervention and until the children are three to three and a half years old.

### 3.6 Step 6: Creation of the assessment plan

The indicators used to evaluate the effect of the intervention outlined in Table 6. The primary outcomes of infants and toddlers with CL/P included the stop consonant inventory, consonant inventory, phonological processes (speech error types), cleft speech characteristics, velopharyngeal function-related quality of life, and intelligibility in context scale. These outcomes will collect and evaluate at six times: baseline, post-treatment, 6-month follow-up, 12-month follow-up, 18-month follow-up and 24-month follow-up.

**Table 6:** The assessment objects, variables, and tools for interventions.

Level	Variable	Instrument
Infants and toddlers with CL/P	Stop consonant inventory Consonant inventory Percentage of consonants correct Development speech characteristics Cleft speech characteristics Velopharyngeal insufficiency-related quality of life Speech Intelligibility	Mandarin Articulation and Phonology Test (45) Velopharyngeal Insufficiency Effects on Life Outcomes instrument (46, 47) Intelligibility in Context Scale (48)
Parents	Parent–child interaction skills Sustainability of parent–child interaction Parent–child interactive environment setting	A video observational analysis
Environment	Family-rearing environment	Family Care Indicators

These outcomes will collect and evaluate at six times: baseline, post-treatment, 6-month follow-up, 12-month follow-up, 18-month follow-up and 24-month follow-up.

## 4 Discussion

This study thoroughly detailed the systematic creation of a family- implemented early intervention aimed at enhancing speech and language skills in Mandarin-speaking infants diagnosed with CL/P. Through the implementation of the established IM approach, known for its effectiveness in planning and developing interventional studies, the researchers developed the intervention in Mandarin-speaking infants and identified the relevant strengths and limitations. This approach thoroughly highlights evidence-based reviews, interviews, stakeholder data, and behavior change theory while incorporating stakeholder input. The groundbreaking nature of this study lies in its original application of the IM approach to benefit Mandarin-speaking infants, simultaneously offering valuable insights for practitioners and researchers on an international scale.

Children learn language through communicative exchanges with their caregivers, initially through vocal and visual cues and later through language use. Parents play a key role in supporting this development by providing prompts and responding to their child’s behavior, encouraging and reinforcing their communicative intentions (40). Infants with CL/P are more susceptible to speech sound disorders and low-frequency pronunciation, leading to a lower speech frequency (41), adversely affecting parent–child interactions and reducing speech and language input quality and quantity. Infants with delayed speech and language development also require more support than usual, as daily parent–child interactions may not be sufficient to facilitate their language acquisition (42). Four factors in parent–child interaction linked to child language development are: the quantity and quantity of interaction, feedback ability, and use of language learning support strategies (24). Various individual and environmental factors may be hurdles for infants with CL/P, leading to a suboptimal natural speech and language environment (13). Intervention programs and encouragement to provide speech and language support before and during the emerging period can help infants with CL/P and delayed speech and language development.

The findings of the problem diagnosis revealed that parents of infants with CL/P lacked the necessary interaction skills and often used inappropriate forms of speech and language while engaging with their infants. This was a significant barrier to achieving speech and language developmental milestones. In addition, parents lacked interest in interaction games, exhibited a poor selection of themes, and showed discontinuity of interactions, which also negatively impacted the infants’ developmental progress. Moreover, the problem diagnosis indicated that many parents struggled in selecting appropriate toys and creating an interaction-rich environment for their infants. Despite the issues outlined in the study, the analysis showed that parents maintained a clear motivation and objective for promoting their infants’ speech and language development. The survey outcomes were vital for developing intervention programs to address these challenges, including the logical model of change.

The ecological system approach, as a theoretical framework, is central to the way IM understands and addresses health problems through intervention (43). We believe that the behavioral treatment plan and proposed outcomes are a component of a multifaceted and interconnected system. In line with this, our program focuses on interaction environment analysis as the cornerstone of our behavioral change strategy, with the parent–child interaction environment and behavior as our primary objective. The technical core of early intervention programs lies in selecting suitable language intervention techniques, teaching them to parents, providing feedback, and confirming their degree of acquisition. This dynamic process allows the language environment for infants with CL/P to be changed and optimized, leading to improved articulation and vocabulary skills.

As the family is a constant factor in children’s lives, a family-centered intervention, which trains parents on effective techniques and concepts, optimizes the speech and language environment of CL/P children. Our intervention approach emphasizes the set-up and use of the communication environment to improve input language. Language modeling, interactive prompting, and phonological emphasis techniques have been adopted for infants with CL/P. To transfer intervention techniques uniformly based on games, therapists display and teach techniques to parents in play-based interactions, which help children learn language in motivating interventions. Subsequently, well-trained parents use these techniques in natural family life, allowing children to participate in daily communication and progress in the crucial period of speech and language development.

One of the main challenges of family-based interventions focusing on parent–child interaction is ensuring that parents receive adequate training and correctly apply the learned techniques. This requires tailored training programs based on individual children’s needs and their parent’s varied emotional, cognitive, and behavioral intentions. To address these challenges, the implementation plan involves face-to-face workshop training sessions between speech/language pathologists and parents to teach them appropriate techniques and individualized video feedback intervention via WeChat based on parent–child interactive behaviors and language input behaviors to provide ongoing support. This approach has led to additional benefits, as individual training is a more satisfying solution for parents, resulting in higher compliance and better clinical outcomes (31, 44). However, therapists are in higher demand due to the length of time they need to dedicate to their work, which requires more human input.

To ensure the equitable delivery of the intervention, we will establish an internal quality control and training protocol. This will involve the creation of an implementation toolkit designed to enhance parental participation in early intervention programs, thereby facilitating standardization and quality assurance. The toolkit comprises an implementation plan and materials for the early intervention program, workshop lecture points, video feedback guidelines, and a checklist of subgoals for parent behavior during video feedback sessions. The integrity of the intervention will be monitored at regular intervals by a study coordinator to ensure consistent adherence to the established standards.

This study had three strengths. Firstly, a parent-implemented early intervention program aimed at enhancing speech and language skills for infants and toddlers with CL/P was developed utilizing the IM tools. It fills the lack of research on the early interventions for Mandarin-speaking infants and toddlers. Second, the multi-level goal setting in the IM strategy, which includes outcomes, performance objectives, and change objectives, makes complex parent–child interactive intervention both systematic and individualized. Third, the IM approach helped us organize intervention techniques and their transmission, confirming the implementation of these techniques and forming a behavioral loop. This enabled us to develop a feasible intervention that can be integrated into existing child health delivery channels. We found many strengths in using the IM approach, particularly the explicit inclusion of theory, evidence, and guidance on developing the intervention in partnership with local stakeholders.

There were several limitations to the study. Firstly, the non-randomized intervention study design has inherent limitations, but a randomized control trial presents ethical concerns because the aim of the ‘intervention’ is to implement best practice elements. Second, the low incidence of CL/P, combined with the critical impact of age on language development, limited the sample size due to the narrow age range. Finally, although the recruitment of participants was conducted at a center providing sequential treatment for patients with CL/P from all over the country, particularly in the northwestern areas, this single-center study could partially constrain the diversity of the participants and the generalizability of the findings. These findings regarding the diagnosis of interactional problems hold significant socio-cultural and linguistic relevance. To enhance the applicability of the intervention program, future problem analyses and intervention trials should be conducted and validated across a broader range of socio-economic and geographic contexts. Additionally, in the intervention implementation phase, we will further examine various aspects such as their reach, deliverability, uptake, success, and generalizability to further understand their strengths and limitations.

## 5 Conclusion

A parent-implemented early intervention was developed, including the following specific steps: health education within the hospital, 9 days of an online reading program in WeChat groups, face-to-face standardized training workshops, and individualized video feedback therapy. The intervention aims to support the adoption of parent–child interactive intervention by increasing knowledge, skill, and motivation and targeting key individuals, interpersonal skills, and contexts. The goal of this step is to diagnose the problems and design program implementation strategies. If it turned out to be feasible and effective by our further study, the intervention and implementation strategies will be made available to other hospitals. It may help to overcome barriers of insufficient professional services provided by speech–language therapists/pathologists in China.

## Data Availability

The raw data supporting the conclusions of this article will be made available by the authors, without undue reservation.
